# DNA mismatch repair gene *MSH6* implicated in determining age at natural menopause

**DOI:** 10.1093/hmg/ddt620

**Published:** 2013-12-19

**Authors:** John R.B. Perry, Yi-Hsiang Hsu, Daniel I. Chasman, Andrew D. Johnson, Cathy Elks, Eva Albrecht, Irene L. Andrulis, Jonathan Beesley, Gerald S. Berenson, Sven Bergmann, Stig E. Bojesen, Manjeet K. Bolla, Judith Brown, Julie E. Buring, Harry Campbell, Jenny Chang-Claude, Georgia Chenevix-Trench, Tanguy Corre, Fergus J. Couch, Angela Cox, Kamila Czene, Adamo Pio D'adamo, Gail Davies, Ian J. Deary, Joe Dennis, Douglas F. Easton, Ellen G. Engelhardt, Johan G. Eriksson, Tõnu Esko, Peter A. Fasching, Jonine D. Figueroa, Henrik Flyger, Abigail Fraser, Montse Garcia-Closas, Paolo Gasparini, Christian Gieger, Graham Giles, Pascal Guenel, Sara Hägg, Per Hall, Caroline Hayward, John Hopper, Erik Ingelsson, kConFab investigators, Sharon L.R. Kardia, Katherine Kasiman, Julia A. Knight, Jari Lahti, Debbie A. Lawlor, Patrik K.E. Magnusson, Sara Margolin, Julie A. Marsh, Andres Metspalu, Janet E. Olson, Craig E. Pennell, Ozren Polasek, Iffat Rahman, Paul M. Ridker, Antonietta Robino, Igor Rudan, Anja Rudolph, Andres Salumets, Marjanka K. Schmidt, Minouk J. Schoemaker, Erin N. Smith, Jennifer A. Smith, Melissa Southey, Doris Stöckl, Anthony J. Swerdlow, Deborah J. Thompson, Therese Truong, Sheila Ulivi, Melanie Waldenberger, Qin Wang, Sarah Wild, James F Wilson, Alan F. Wright, Lina Zgaga, ReproGen Consortium, Ken K. Ong, Joanne M. Murabito, David Karasik, Anna Murray

**Affiliations:** 1 University of Exeter Medical School, Exeter, UK,; 2 Wellcome Trust Centre for Human Genetics, University of Oxford, Oxford, UK,; 3 Department of Twin Research and Genetic Epidemiology, King's College London, London, UK,; 4 Medical Research Council (MRC) Epidemiology Unit, Institute of Metabolic Science, Addenbrooke's Hospital, Cambridge, UK,; 5 Hebrew SeniorLife Institute for Aging Research and Harvard Medical School, Boston, MA, USA,; 6 Molecular and Integrative Physiological Sciences Program, Harvard School of Public Health, Boston, MA, USA,; 7 Division of Preventive Medicine, Brigham and Women's Hospital, 900 Commonwealth Avenue East, Boston MA 02215, USA,; 8 Harvard Medical School, Boston, MA, USA,; 9 The National Heart Lung and Blood Institute's Framingham Heart Study, Framingham, MA, USA,; 10 NHLBI Cardiovascular Epidemiology & Human Genomics Branch, Bethesda, MD, USA,; 11 Institute of Genetic Epidemiology,; 12 Institute of Epidemiology II and; 13 Research Unit of Molecular Epidemiology, Helmholtz Zentrum München – German Research Center for Environmental Health, Neuherberg, Germany,; 14 Lunenfeld-Tanenbaum Research Institute, Mount Sinai Hospital, Toronto, ON, Canada,; 15 Department of Molecular Genetics, University of Toronto, Toronto, ON, Canada,; 16 Department of Genetics, Queensland Institute of Medical Research, Brisbane, QLD, Australia,; 17 Department of Epidemiology, Tulane University, New Orleans, LA, USA,; 18 Department of Medical Genetics, University of Lausanne, Lausanne, Switzerland,; 19 Swiss Institute of Bioinformatics, Lausanne, Switzerland,; 20 Department of Clinical Biochemistry and the Copenhagen General Population Study, Herlev Hospital, Copenhagen University Hospital, Copenhagen, Denmark,; 21 Department of Breast Surgery, Herlev Hospital, Copenhagen University Hospital, Copenhagen, Denmark,; 22 Centre for Cancer Genetic Epidemiology and Department of Public Health and Primary Care, University of Cambridge, Cambridge, UK,; 23 Department of Paediatrics, University of Cambridge, Cambridge, UK,; 24 Centre for Population Health Sciences, University of Edinburgh, EdinburghEH8 9AG, UK,; 25 Division of Cancer Epidemiology, German Cancer Research Center, Heidelberg, Germany,; 26 Departments of Laboratory Medicine and Pathology, and Health Science Research,; 27 Department of Health Sciences Research, Mayo Clinic, Rochester, MN, USA,; 28 CR-UK/YCR Sheffield Cancer Research Centre, Department of Oncology, University of Sheffield, UK,; 29 Medical Epidemiology and Biostatistics, Karolinska Institutet, Stockholm, Sweden,; 30 Department of Oncology-Pathology, Karolinska Institutet, Stockholm, Sweden,; 31 Institute for Maternal and Child Health, IRCCS ‘Burlo Garofolo’, University of Trieste, Trieste, Italy,; 32 Centre for Cognitive Ageing and Cognitive Epidemiology,; 33 Department of Psychology and; 34 MRC Human Genetics Unit, Institute of Genetics and Molecular Medicine, University of Edinburgh, Edinburgh, UK,; 35 Division of Psychosocial Research and Epidemiology and; 36 Division of Molecular Pathology, The Netherlands Cancer Institute, Amsterdam, The Netherlands,; 37 Department of General Practice and Primary Health Care, University of Helsinki, Helsinki, Finland,; 38 National Institute for Health and Welfare, Helsinki, Finland,; 39 Folkhälsan Research Centre, Helsinki, Finland,; 40 University Central Hospital, Unit of General Practice, Helsinki, Finland,; 41 Vasa Central Hospital, Vasa, Finland,; 42 Divisions of Endocrinology, Children's Hospital, Boston, MA, USA,; 43 Broad Institute, Cambridge, MA, USA,; 44 Estonian Genome Center, University of Tartu, 51010Tartu, Estonia,; 45 Department of Gynecology and Obstetrics, University Hospital Erlangen, Friedrich-Alexander University Erlangen-Nuremberg, Erlangen, Germany,; 46 Division of Cancer Epidemiology & Genetics, National Cancer Institute, Maryland, USA,; 47 School of Social and Community Medicine, MRC Centre for Causal Analyses in Translational Epidemiology, University of Bristol, Bristol, UK,; 48 Divisions of Breast Cancer Research and of Genetics and Epidemiology, and the Breakthrough Breast Cancer Research Centre, The Institute of Cancer Research, London, UK,; 49 Centre for Molecular, Environmental, Genetic and Analytic Epidemiology, Melbourne School of Population Health, The University of Melbourne, Melbourne, VIC, Australia,; 50 Cancer Epidemiology Centre, The Cancer Council Victoria, Melbourne, VIC, Australia,; 51 Environmental Epidemiology of Cancer, Inserm U1018, Villejuif, France,; 52 Department of Medical Sciences, Molecular Epidemiology and Science for Life Laboratory, Uppsala University, Uppsala, Sweden,; 53 Institute of Genetics and Molecular Medicine, University of Edinburgh, Western General Hospital, EdinburghEH4 2XU, UK,; 54 Peter MacCallum Cancer Centre, Melbourne, VIC, Australia,; 55 Department of Epidemiology, University of Michigan, Ann Arbor, MI, USA,; 56 Institute of Behavioural Science, University of Helsinki, Helsinki, Finland,; 57 School of Women's and Infants’ Health, University of Western Australia, Australia,; 58 Department of Public Health, Faculty of Medicine, University of Split, Croatia,; 59 Department of Obstetrics and Gynecology, University of Tartu, 51014 Tartu, Estonia,; 60 Competence Centre on Reproductive Medicine and Biology, 50410 Tartu, Estonia,; 61 Department of Pediatrics and Rady Children's Hospital, University of California San Diego, La Jolla, CA 92093, USA,; 62 Genetic Epidemiology Laboratory, Department of Pathology, The University of Melbourne, Melbourne, VIC, Australia,; 63 Department of Obstetrics and Gynaecology, Campus Grosshadern, Ludwig-Maximilians-University, Munich, Germany,; 64 Institute for Maternal and Child Health, IRCCS ‘Burlo Garofolo’, Trieste, Italy,; 65 Section of General Internal Medicine, Department of Medicine, Boston University School of Medicine, Boston, MA, USA

## Abstract

The length of female reproductive lifespan is associated with multiple adverse outcomes, including breast cancer, cardiovascular disease and infertility. The biological processes that govern the timing of the beginning and end of reproductive life are not well understood. Genetic variants are known to contribute to ∼50% of the variation in both age at menarche and menopause, but to date the known genes explain <15% of the genetic component. We have used genome-wide association in a bivariate meta-analysis of both traits to identify genes involved in determining reproductive lifespan. We observed significant genetic correlation between the two traits using genome-wide complex trait analysis. However, we found no robust statistical evidence for individual variants with an effect on both traits. A novel association with age at menopause was detected for a variant rs1800932 in the mismatch repair gene *MSH6* (*P* = 1.9 × 10^−9^), which was also associated with altered expression levels of *MSH6* mRNA in multiple tissues. This study contributes to the growing evidence that DNA repair processes play a key role in ovarian ageing and could be an important therapeutic target for infertility.

## INTRODUCTION

Female reproductive lifespan starts just prior to menarche, the onset of the first menstrual bleed and finishes when oocyte supply becomes exhausted at menopause. Both processes are governed by genetic and non-genetic factors and the timing of these events is associated with multiple adverse health outcomes, including breast cancer, cardiovascular disease, osteoporotic fractures and infertility (1). Recent genome-wide association studies (GWAS) have identified 32 loci involved in age at menarche (2) and 17 with age at natural menopause (3): however none of the variants identified to date overlap. Epidemiological studies also do not strongly support a role for overlapping aetiology in the processes governing timing of menarche and menopause (4–7). Age at menarche has decreased significantly in recent history and this has been thought to be largely due to increased levels of childhood obesity (8–10). The role of adiposity in regulating menarche timing is supported by genetic studies which have reported that many genes involved in the regulation of fat mass are also associated with timing of menarche (11–13). Secular changes in menopause age are more controversial with individual studies reporting conflicting findings (14,15). The correlation between ages at menopause and menarche is also controversial, but larger studies show a modest correlation between the two phenotypes (16) and as both events involve the same organ system, it is conceivable that there are common physiological processes involved, which may be influenced by genetic and environmental factors.

The length of reproductive lifespan has been associated with several adverse health outcomes, particularly breast cancer. Genes involved in regulating reproductive lifespan in humans have not been described to date. There are two ways in which reproductive lifespan can be altered: either total length, or it can be temporally shifted, either earlier or later. These shifts in lifespan would not be detected if the outcome measured was the length of reproductive lifespan. It is possible that both menarche and menopause could occur early, yet the time period between the two events could be normal. In order to capture the features of this phenotype and investigate the underlying genetic aetiology, we used a bivariate GWAS method to identify genetic loci associated with both age at menopause and menarche in either direction. This study incorporated GWAS data from the *ReproGen* consortium meta-analyses of 87 802 individuals for menarche and 38 968 individuals for menopause (2,17).

## RESULTS

### Genetic correlation between traits

We performed a restricted maximum likelihood (REML) bivariate analysis (18,19) within the *Women's Genome Health Study* (total sample *N* = 21 505) to test for genome-wide genetic correlation between timing of menarche and menopause. Using 329 966 autosomal SNPs we observed a positive correlation of *r*_genetic_ = 0.138 (*P* = 0.04, s.e 0.068). This result remained similar after adjustment for the top 10 principal components of population stratification.

### Bivariate meta-analysis

The bivariate meta-analysis for menarche and menopause generated two signals with genome-wide significant *P* values <5 × 10^−8^ and a further four independent signals with *P* values <1 × 10^−7^ (Table 1). We assessed the association with each of the individual traits of the top bivariate signal plus SNPs in linkage disequilibrium (LD) with the best SNP. Of the six top hits, for four signals either the top SNP, or a SNP in LD with the top SNP (hapmap *r*^2^ > 0.05), had been previously identified in the GWAS for one of the traits individually. The strongest bivariate signal was near *GAB2*, a known locus for menarche (2) and SNPs near *FSHB*, *SYCP2L* and *PRRC2A* were known loci for menopause (3). There were two signals near *RPAIN* and *MSH6*, which had not been previously reported for either trait and had *P* values of 1 × 10^−7^ and 3 × 10^−7^, respectively in the bivariate analysis. We did not have sufficiently robust statistical evidence for any locus being associated with both menopause and menarche and thus influencing reproductive lifespan. An ingenuity pathway analysis of the top six signals (www.ingenuity.com) found an enrichment in the ovarian cancer signalling pathway (*P* = 7.67 × 10^−4^), with three of the six genes closest to the top variants being in that pathway (*FSHB*, *GAB2* and *MSH6*).

**Table 1.** Results of bivariate analysis

Region	Gene	SNP	Known region?	*r*^2^ with known GWAS SNP	Ref allele	Bivariate, *P*-value	Effect on reproductive lifespan
11q14.1	GAB2	rs11603112	Menarche (2)	0.82	A	4.13 × 10^−10^	Right shift
11p14.1	FSHB	rs11031002	Menopause (3)	0.76	A	1.63 × 10^−09^	Right shift
6p24.2	SYCP2L	rs9467921	Menopause (3)	0.08	C	9.82 × 10^−08^	Lifespan increase
6p21.32	*PRRC2A*	rs2471980	Menopause (3)	0.45	C	1.17 × 10^−07^	Left shift
17p13.2	*RPAIN*	rs4790770	–	–	A	1.24 × 10^−07^	Lifespan decrease
2p16.3	*MSH6*	rs1800932	–	–	A	3.27 × 10^−07^	Left shift

Effect on reproductive lifespan gives the effect directions for both menarche and menopause associations; right shift = menarche increasing allele is menopause increasing allele (and vice versa for left shift), reproductive lifespan increase = menarche decreasing allele same as menopause increasing allele (vice versa for reproductive lifespan decrease). Known regions are loci that have been identified in previous GWAS efforts for the individual traits.

### Replication of top signals in individual traits

**Figure 1.**
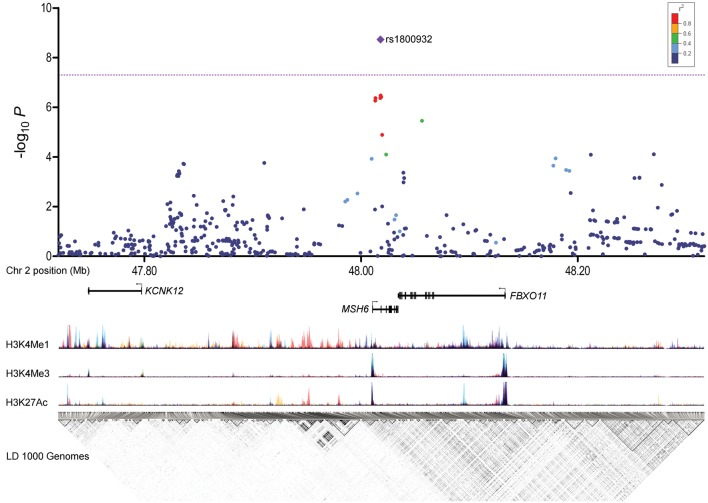
Regional plot illustrating the strength of association (−log_10_ *P*) versus hg19 position. The purple diamond represents the lead SNP in the combined analysis of replication and discovery data. Other dots, coloured according to the degree of pairwise linkage disequilibrium, represent single SNP test statistics for discovery stage only, including rs1800932, which was the SNP with lowest *P* value in the discovery analysis. The lower panels show structures of genes; layered ENCODE histone modification marks (H3K4Me1 which marks enhancers; H3K4Me3 which marks promoters and H3K27Ac which marks active regulatory regions) and linkage disequilibrium pairwise correlation (*r*^2^) derived from the CEU population in the 1000 Genomes Project, where white corresponds to *r*^2^ = 0 and black to *r*^2^ = 1.

In order to increase our power to detect SNPs associated with both traits, we increased our sample size for the top six loci. We tested the top 6 signals in additional *in silico* replication cohorts, including up to 28 470 individuals from 22 studies for menarche and up to 19 851 individuals from 22 studies for menopause (Table 2). One of the six bivariate SNPs from the discovery analysis reached genome-wide significance in the combined meta-analysis for menopause alone (rs1800932, *P* = 2 × 10^−9^). This variant was in the *MSH6* gene on chromosome 2 and was associated with a 1.3 months (se = 0.38) reduction in menopause age per common allele in the replication cohorts alone (allele frequency = 0.83) (Fig. 1).

**Table 2.** Follow-up of top six bivariate signals for association with individual traits

Gene	Top bivariate SNP	Menarche (*P*)	Menopause (*P*)	Top Menarche SNP in region						Top Menopause SNP in region					
				SNP	*r*^2^	Discovery (*P*)	Replication (*P*)	Combined (*P*)	*N*	SNP	*r*^2^	Discovery (*P*)	Replication (*P*)	Combined (*P*)	*N*
*GAB2*	rs11603112	5.79E–10	1.05E–04	rs10899489	0.82	2.41E–10	0.02	4.62E−11	116 268	rs2450129	0.84	4.62E−05	0.15	1.94E−04	48 716
*FSHB*	rs11031002	4.56E–05	3.52E−08	rs10835649	0.71	8.17E−06	0.71	3.71E−04	116 268	rs12294104	0.76	1.63E−08	1.89E−04	6.16E−11	48 717
*SYCP2L*	rs9467921	2.56E−03	3.93E−07	rs9467921	−	2.56E-03	0.88	8.50E−03	116 267	rs2153157	0.08	9.40E−11	0.07	4.02E−11	48 715
*PRRC2A*	rs2471980	2.20E-04	1.18E−06	rs660594	0.13	1.81E−04	0.11	5.14E−05	116 264	rs1046089	0.45	1.31E−13	2.96E−04	2.59E−14	48 708
*RPAIN*	rs4790770	7.48E−06	7.79E−05	rs8074617	1.00	7.00E−06	0.60	4.30E-05	116 266	rs4790772	0.88	8.78E−06	0.15	6.24E−05	48 716
*MSH6*	rs1800932	4.89E−03	3.35E−07	rs3136247	1.00	4.41E−03	0.60	3.44E−02	116 267	rs1800932	−	3.35E−07	0.0004	1.87E−09	58 812

For menarche 11 SNPs had a direction of effect in the replication samples consistent with the discovery samples and 5 did not. For menopause, 15 were consistent and 1 was not.

### Functional significance of novel variant for age at menopause

We investigated the functional significance of the novel locus associated with age at menopause by determining whether the top variant, or SNPs in LD with it, were associated with expression levels of any genes in the genome. We accessed data from four tissue types (monocytes, whole blood, liver and lung) and in all tissues rs1800932 or rs3136247 (*r*^2^ = 0.95) was associated with expression of *MSH6* (*P* = 3.9 × 10^−6–^3.1 × 10^−20^). In monocytes, whole blood and liver, rs3136247 was the SNP most strongly associated with expression of *MSH6*, but in lung tissue the best SNP was rs2047681 (*r*^2^ = 0.6 with rs3136247).

### SNPs in LD with top bivariate signals

In addition to the top bivariate SNP, we chose to also replicate the strongest SNP from each individual trait GWAS, which was in LD (hapmap *r*^2^ > 0.8) with the lead bivariate SNP (Table 2). In the individual menarche GWAS five independent SNPs in LD with a bivariate signal were more strongly associated with menarche than the lead bivariate SNP. Following the replication stage, none of the five SNPs reached genome-wide significance in the combined analysis of discovery and replication data, but statistical evidence increased for a known menopause locus in *PRRC2A* being associated with age at menarche following replication, with the *P* value strengthening from 2 × 10^−4^ to 5 × 10^−5^. In the univariate menopause GWAS five SNPs in LD with top bivariate SNPs gave a stronger association and were taken forward for replication, but the only SNPs that gave a stronger association following replication were the three previously known menopause signals.

## DISCUSSION

Female reproductive lifespan starts at menarche and ends at menopause and we therefore performed a bivariate analysis of the two traits. Two signals reached genome-wide significance in this analysis, but these were both heavily driven by low *P* values in one of the individual traits. *GAB2* is a known signal for menarche and *FSHB* for menopause (2,3). We sought to improve the statistical association for the second trait by *in silico* replication, but for both variants the statistical evidence got weaker. We also took four bivariate signals forward for additional replication, which were just below the genome-wide significance threshold, but none were robustly associated in both traits. The most promising bivariate signal after replication was associated with a left shift in reproductive lifespan and was in the HLA III region on chromosome 6 (near *PRRC2A* gene), which is a known signal for menopause and reached *P* = 5 × 10^−5^ for menarche, following replication. This genomic region has also been associated with other phenotypes from GWAS scans, e.g. type 1 diabetes, multiple sclerosis and lupus (http://www.genome.gov/gwastudies/). This would be a good candidate to follow-up in additional cohorts with menarche data and if substantiated would provide evidence for an immunological pathway involved in both phenotypes. Despite finding no individual locus associated with both age at menarche and age at menopause, the positive although modest genetic correlation (*r*_genetic_ = 0.138) suggests that common genetic loci do exist, likely with very subtle effects. Our pathway analysis also suggests that there be one or two pathways of shared aetiology between the two traits, with many others uniquely involved.

Future studies could include increasing the sample size in the discovery meta-analysis or replication of the top signals in additional cohorts. This is important since most of our participating studies were Caucasian, therefore other ethnic groups need to be similarly analysed. Studies are ongoing which include individuals of multiple ethnicities, which should narrow down the association further. Also, other than adjusting for birth year in menarche analysis, we did not correct for environmental factors which may influence reproductive life span (such as early obesity or adult-age smoking). A further limitation of our study is that the cohorts included in the analysis were predominantly the same as those used in the meta-analyses for the individual traits (2,3). Thus, finding that four of the six top hits for reproductive lifespan had been reported previously for one of the individual traits, was not necessarily surprising, as these had very low *P* values in the original analysis. One of the limitations of the bivariate meta-analysis is that very strong signals in individual traits can drive the bivariate statistic over the *P* < 5 × 10-8 threshold. The method is therefore best applied to signals that are sub-genome-wide significant in individual traits, or to use in pathway analyses than can highlight biological processes common to both traits.

We identified a novel variant associated with age at natural menopause which is a synonymous exonic SNP in the *MSH6* gene on chromosome 2. The SNP had a minor allele frequency of ∼18% and the rare allele increased menopause age by ∼1.3 months per allele. MSH6 heterodimerizes with MSH2 to form the MutS alpha protein complex, which is involved in mismatch repair (MMR), predominantly of single base mismatches and small dinucleotide insertion/deletion loops (20). It forms a complex with MutL alpha which is a heterodimer of PMS1 and MLH1. Germline mutations in *MSH6*, *MSH2* and *MLH1* have been associated with hereditary non-polyposis colorectal cancer (HNPCC) (21). *MSH6* mutations are rarer than mutations in the other two genes, being found in 10–20% of HNPCC cases, who often have an atypical presentation and increased predisposition to endometrial cancer (22,23). No role for *MSH6* in reproductive ageing has been described previously, but other genes involved in DNA repair were identified in the recent GWAS for age at menopause, including *EXO1*, *UIMC1*, *MCM8 and POLG(3).* EXO1 has 5′–3′ exonuclease activity and interacts with several of the MMR proteins, including MSH2, MLH1, MSH3, PCNA and WRN for its role in MMR and recombination (24). UIMC1 recruits BRCA1 to DNA damage sites and initiates G2/M checkpoint control (25). MCM8 is a member of a family of DNA replication complex proteins and is thought to have a role in meiotic double-strand break repair (26). Finally, POLG is responsible for the replication and repair of the mitochondrial genome (27). Thus, variation in DNA repair processes, including single nucleotide, double-strand and mitochondrial DNA repair, appear to play a crucial role in determining age at natural menopause. A recent paper showed accumulation of double-strand DNA breaks in human follicles with age, with concomitant downregulation of key DNA repair genes *BRCA1*, *MRE11*, *Rad51* and *ATM*, providing evidence that these processes play a functional role in ovarian ageing (28). It has also been reported that carriers of germline mutations in MMR genes, namely *BRCA1* and *BRCA2* are at increased risk of early menopause (29–32). We demonstrated that rs1800932 is an expression quantitative trait locus for *MSH6*, with the rarer allele being associated with increased levels of mRNA. Thus, the lower expression of *MSH6* is associated with earlier menopause, consistent with the work of Titus *et al*. (28), where downregulation of DNA repair genes was associated with ovarian ageing. The effect of the *MSH6* variant on menopause age is relatively small and only explains a small proportion of the variance in menopause age. There are thus likely to be many more undiscovered genetic variants responsible for determining age at natural menopause.

The mechanism by which DNA repair influences timing of menopause is yet to be determined, but it is conceivable that damage to DNA of meiotic cells, if not repaired effectively, would result in the activation of apoptotic pathways to prevent transmission of potentially deleterious mutations to offspring. It is known that in non-meiotic cells DNA damage results in increased apoptosis and thus it is conceivable that similar processes affect germ cells. Mouse models of the *BRCA1* MMR gene have marked depletion of germ cells (33). Oocytes are lost throughout female life until menopause, predominantly by apoptosis. Any increase in apoptosis would therefore be expected to exhaust the oocyte pool prematurely and result in earlier menopause. We have described a novel association between a key DNA MMR gene and age at menopause, implicating MMR as a key process involved in determining reproductive lifespan.

## MATERIALS AND METHODS

### Estimation of genetic correlation between menarche and menopause

We used our largest individual study the *Women's Genome Health Study* (WGHS) (total sample *N* = 23 294) to test for genome-wide genetic correlation between timing of menarche and menopause. The total joint contribution of common SNPs to both age of menarche and age of menopause in the WGHS was estimated using the REML method implemented in GCTA (18,19). The genetic-related matrix in the WGHS was calculated using 329 966 autosomal SNPs with minor allele frequency >0.01. For analysis of the joint heritability, this matrix was pruned to include only women with relatedness estimate <0.025, leaving a total of 21 505 individuals with age of menarche and 11 025 with age at natural menopause.

### GWA studies for menarche and menopause

Full details of the individual GWAS can be found in the original publications, but briefly: the menarche GWAS included 32 studies, comprising 87 802 women and the menopause GWAS included 22 studies, comprising 38 968 women: all were of White European ancestry. Nineteen studies were included in both GWAS meta-analyses, although the number of women from each study differed (*N* = 71 942 for menarche and *N* = 33 460 for menopause). Women with recalled age at menarche between 9 and 17 years were included in the analysis. Age at natural menopause was defined as the age at the last menstrual period that occurred naturally between the ages of 40 and 60 years. Women were excluded with menopause due to hysterectomy and/or bilateral ovariectomy, or chemotherapy/irradiation, if validated by medical records, and women using HRT before menopause. All studies were approved by local ethics committees and all participants provided written informed consent. Each study performed genome-wide association testing for age at menarche or menopause across ∼2.5 million imputed SNPs, based on linear regression under an additive genetic model. Individual study data were meta-analysed using the METAL software package; genomic control (GC) adjustments were applied. We considered *P*-values of <5 × 10^−8^ to indicate genome-wide significance.

### Discovery bivariate GWAS meta-analysis

We performed multi-phenotype GWAS meta-analyses with aggregate data (*Z* test statistics) from each univariate GWAS meta-analysis (inverse-variance meta-analysis with GC controls, as described above) of age at menarche and natural menopause using our newly developed algorithm, empirical-weighted linear-combined test statistics (eLC) (34,35). Briefly, eLC directly combined correlated test statistics obtained from univariate GWAS meta-analyses with a weighted sum of univariate test statistics to empirically maximize the overall association signals and also to account for the phenotypic correlation between menarche and menopause. Our eLC approach is expressed as

$${S_{{\rm eLC}}} = \sum\limits_1^k {[max(|{T_k}|,c)} ^*|{T_k}|]$$

where *c* is some given non-negative constant. The weight in this new test statistics will be optimally determined by the specific data structure. For instance, when *c* = 0, the test statistics simply reduces into sum of squares of *T_k_*. When *c* is relatively large, equal weight is assigned to each *T_k_.* Ideally, we would like to find an optimal value of *c*, so the ${S_{{\rm eLC}}}$ performs as a linear combination of *T_i_* when under the H_0_; but, under the alternative H_A_, more weight is given to the larger true *T_i_*. The bonafide *P*-value for ${S_{{\rm eLC}}}$ then can be estimated by applying permutation or perturbation techniques. The variance–covariance matrix **Σ** of univariate test statistics using the sample covariance matrix of the test statistics of all SNPs from univariate GWAS analyses as an approximation. **Σ**:

$$\left[ {\matrix{ {\hbox{Var}({Z_1})} & {\hbox{Cov}({Z_1},{Z_2})} \cr {\hbox{Cov}({Z_1},{Z_2})} & {\hbox{Var}({Z_2})} \cr } } \right]$$

where *Z*_1_ consists of unbiased univariate test statistics of all the SNPs for the first trait on genome-wide scale, so does *Z*_2_. On the other hand, **Σ** can be estimated by using generalized least squares from individual-level data. Bivariate *P*-values of <5 × 10^−8^ were considered genome-wide significant with potential pleiotropic effects, except when one of the individual trait *P* values was lower than the bivariate *P* value. The eLC method is implemented in eLX package using C++ and is available at https://sites.google.com/site/multivariateyihsianghsu/.

### Replication strategy

All SNPs with a *P* value of <1 × 10^−7^ in the bivariate analysis were taken forward for replication in 22 additional cohorts with *in silico* data, which included data from the iCOGs meta-analysis, which incorporated 16 individual studies. We also determined for each SNP whether any SNPs in LD with the lead SNP (<1 Mb away and *r*^2^ > 0.8) were more strongly associated with each of the traits in the individual menarche or menopause GWAS studies. There were 10 proxy SNPs with a lower *P* value in the individual trait and these were also taken forward for *in silico* replication. Therefore, in total 16 SNPs were tested in replication cohorts (Supplementary Material, Table S1).

Access to data is available via the GREAT database https://ifar-great.hsl.harvard.edu/ or by contacting the authors directly.

### eQTL analysis

The novel genome-wide associated SNP for age at menopause (rs1800932) and SNPs in LD with this SNP (*r*^2^ > 0.9) were searched against a multi-tissue eQTL database of expression of SNP results (3). In four of the tissues [monocytes (36), blood (37), lung (38) and liver (39)] there was an eQTL for *MSH6* and for three of these (monocytes, blood and liver) the SNP most strongly associated with expression of *MSH6* was our top GWAS SNP or one in strong LD with it (*r*^2^ > 0.95).

## SUPPLEMENTARY MATERIAL

Supplementary Material is available at *HMG* online.

## FUNDING

J.R.B.P is a Sir Henry Wellcome Postdoctoral Research Fellow (092447/Z/10/Z). D.A.L. and A.F. work in a centre that receives infrastructure funding from the UK Medical Research Council (G0600705) and A.F. is funded by a UK Medical Research Council Post-doctoral research fellowship (0701594). Further information on funding for individual studies is provided in supplementary information. Funding to pay the Open Access publication charges for this article was provided by the Wellcome Trust.

## Supplementary Material

Supplementary Data
