# Supplementary material for: DNA mismatch repair gene MSH6 implicated in determining age at natural menopause
Source: Hum Mol Genet. 2013 Dec 19;23(9):2490–7. doi: 10.1093/hmg/ddt620 (PMC3976329; doi:10.1093/hmg/ddt620)
Supplement: Supplementary Data [file supp_23_9_2490__index.html]

DNA mismatch repair gene MSH6 implicated in determining age at natural menopause — DNA mismatch repair gene MSH6 implicated in determining age at natural menopause — DNA mismatch repair gene MSH6 implicated in determining age at natural menopause — Supplementary Data 

# DNA mismatch repair gene *MSH6* implicated in determining age at natural menopause

## Supplementary Data

Supplementary Data

**Files in this Data Supplement:**

- Supplementary Data - Docx file
