# Supplementary material for: DNA mismatch repair gene MSH6 implicated in determining age at natural menopause
Source: Hum Mol Genet. 2013 Dec 19;23(9):2490–7. doi: 10.1093/hmg/ddt620 (PMC3976329; doi:10.1093/hmg/ddt620)
Supplement: Supplementary Data [file supp_ddt620_ddt620supp.docx]

# Supplementary Table. Summary data for replication cohorts

**Author contributions**:

All authors contributed to the writing and review of the manuscript.

Writing group: JRBP, Y-HH, DIC, DJ, CE, KOO, JM, DK, AM

Meta-analysis and follow-up analyses: JRBP, Y-HH, DIC, ADJ

Oversight of contributing cohorts: JRBP, CE, LS, KOO, AM

Coordination of replication studies: EA, ILA, GSB, SB, SEB, MJB, JB, HC, JC-C, GC-T, TC, FJC, AC, KC, APdA, GD, IJD, JDD, DFE, EME, JGE, TE, PF, JDF, HF, AF, MG-C, PG, CG, GG, PG, SH, PH, CH, JH, EI, SLRK, KK, JAK, JL, DAL, PM, SM, JAM, JEO, CEP, OP, IR, AR, IR, AR, AJS, MS, MS, ENS, JAS, MKS, DS, AS, DJT, TT, SU, MW, QW, SW, JFW, AW, LZ

# REPROGEN CONSORTIUM MEMBERS

Albert V. Smith, Alice M Arnold, André G. Uitterlinden, Andrea Burri, Andrea D. Coviello, Andres Metspalu, Angela Döring, Anna Murray, Barbara McKnight, Barbara Thorand, Bjarke Feenstra, Caroline Hayward, Cathy E. Elks, Christian Gieger, Chunyan He, Craig E. Pennell, Daniel Evans, Daniel F. Gudbjartsson, Daniel I. Chasman, Daniel L. Koller, Daniela Toniolo, David J. Hunter, David Karasik, Doris Stoeckl, Dorret I. Boomsma, Douglas P. Kiel, Eleonora Porcu, Elisabeth Widen, Elizabeth A. Streeten, Enda M. Byrne, Erik Ingelsson, Eva Albrecht, Felix Day, Francesco Cucca, Frank Geller, Gonneke Willemsen, Guangju Zhai, Gudny Eiriksdottir, Heather A. Boyd, Henry Völzke, James F. Wilson, Jenny A.Visser, Joanne M. Murabito, John R.B. Perry, Joop S.E. Laven, Jouke-Jan Hottenga, Julie Marsh, Kathryn L. Lunetta, Ken K. Ong, L. Fernandez Rhodes, Laura Crisponi, Laura J. Bierut, Laura M Yerges-Armstrong, Leslie Raffel, Lina Zgaga, Linda Broer, Lisette Stolk, Maja Barbalic, Manuela Uda, Massimo Mangino, Melissa E Garcia, Melissa Wellons, Michael J. Econs, Michael N. Weedon, Najaf Amin, Nicholas G. Martin, Nicole M. Warrington, Nora Franceschini, Ozren Polasek, Paolo Gasparini, Patrick F. McArdle, Patrick Sulem, Peng Lin, Petra HM Peeters, Pio d'Adamo, Sarah E Medland, Serena Sanna, Shelia Ulivi, Tamara B. Harris, Tanguy Corre, Tim D. Spector, Tõnu Esko, Unnur Thorsteinsdottir, Yvonne V Louwers.

# EXTENDED ACKNOWLEDGMENTS FOR REPLICATION STUDIES

**ALSPAC:** We are extremely grateful to all of the families who took part in this study, the midwives for recruiting them, and the whole ALSPAC team, which includes interviewers, computer and laboratory technicians, clerical workers, research scientists, volunteers, managers, receptionists and nurses. The UK Medical Research Council and Wellcome Trust (092731), together with the University of Bristol, provide core support for the ALSPAC study.

**BHS:** ENS was supported in part by NIH/NCRR Grant Number UL1 RR025774. The BHS was supported by grants HD-061437 and HD-062783 from the National Institute of Child Health and Human Development, and AG-16592 from the National Institute on Aging.

**CoLaus:** The CoLaus study received financial contributions from GlaxoSmithKline, the Faculty of Biology and Medicine of Lausanne, and the Swiss National Science Foundation (33CSCO-122661). The authors thank Peter Vollenweider and Dawn Waterworth, Co-PIs of the CoLaus study. Special thanks to Murielle Bochud, Yolande Barreau, Mathieu Firmann, Vladimir Mayor, Anne-Lise Bastian, Binasa Ramic, Martine Moranville, Martine Baumer, Marcy Sagette and Jeanne Ecoffey.

# EGCUT: EGCUT received targeted financing from Estonian Government SF0180142s08, Center of Excellence in Genomics (EXCEGEN) and University of Tartu (SP1GVARENG). ASa received support from SF0180044s09 and EU30020.We acknowledge EGCUT technical personnel, especially Mr V. Soo and S. Smit. Data analyzes were carried out in part in the High Performance Computing Center of University of Tartu.

**CROATIA VIS, KORCULA and SPLIT:** The VIS, KORCULA and SPLIT studies were funded by grants from the Medical Research Council (UK), European Commission Framework 6 project EUROSPAN (Contract No. LSHG-CT-2006-018947) and Republic of Croatia Ministry of Science, Education and Sports research grants to I.R. (108-1080315-0302). We would like to acknowledge the staff of several institutions in Croatia that supported the field work, including but not limited to The University of Split and Zagreb Medical Schools, Institute for Anthropological Research in Zagreb and Croatian Institute for Public Health. The SNP genotyping for the VIS cohort was performed in the core genotyping laboratory of the Wellcome Trust Clinical Research Facility at the Western General Hospital, Edinburgh, Scotland. The SNP genotyping for the KORCULA cohort was performed in Helmholtz Zentrum Munchen, Neuherberg, Germany. The SNP genotyping for the SPLIT cohort was performed by AROS Applied Biotechnology, Aarhus, Denmark.

**GENOA:** Support for the Genetic Epidemiology Network of Arteriopathy (GENOA) was provided by the National Heart, Lung and Blood Institute of the National Institutes of Health (HL054464, HL054457, HL054481, and HL087660). Genotyping was performed at the Mayo Clinic (Stephen Turner, Mariza de Andrade, Julie Cunningham) and was made possible by the University of Texas Health Sciences Center (Eric Boerwinkle, Megan L. Grove-Gaona). We would also like to thank the families that participated in the GENOA study.

**HBCS:** We thank all study participants as well as everybody involved in the Helsinki Birth Cohort Study. Helsinki Birth Cohort Study has been supported by grants from the Academy of Finland, the Finnish Diabetes Research Society, Folkhälsan Research Foundation, Novo Nordisk Foundation, Finska Läkaresällskapet, Signe and Ane Gyllenberg Foundation, University of Helsinki, European Science Foundation (EUROSTRESS), Ministry of Education, Ahokas Foundation, Emil Aaltonen Foundation, Juho Vainio Foundation, and Wellcome Trust (grant number WT089062).

**INGI-CARLANTINO:** We are very grateful to the municipal administrators for their collaboration on the project and for logistic support. We would like to thank all participants to this study. We thank Angela D’Eustacchio and Anna Morgan for technical support.

**INGI-FVG:** We are very grateful to the municipal administrators for their collaboration on the project and for logistic support. We would like to thank all participants to this study. The study was supported by Regione FVG (L.26.2008). We thank Anna Morgan and Angela D’Eustacchio for technical support.

**INGI – Val Borbera:** We thank the inhabitants of the villages in Val Borbera who have made this study possible. We thank the Val Borbera municipalities, the Tortona and Genova archidiocese and the ASL 22 of Novi Ligure (Alessandria) for support. We are indebted to Mrs Maria Rosa Biglieri for help in the clinical evaluation of the population. We acknowledge the collaboration of the MDs from the School of Internal Medicine and Cardiology of the San Raffaele Hospital and of the local doctors. We especially thank Dr. Diego Sabbi for many suggestions and great support. The research was funded by grants of the Italian Health Ministry (RF-FSR-2007-647201) by Fondazione Compagnia di San Paolo, Fondazione Cassa di Risparmio di Alessandria and Telethon Foundation Onlus Rome. Health Ministry project RF-FSR-2007-647201, Fondazione Compagnia di San Paolo. Fondazione Cassa di Risparmio di Alessandria and Telethon Foundation Onlus Rome.

**iCOGS:** This study would not have been possible without the contributions of the following: Per Hall (COGS); Douglas F. Easton, Paul Pharoah, Kyriaki Michailidou, Manjeet K. Bolla, Qin Wang (BCAC), Andrew Berchuck (OCAC), Rosalind A. Eeles, Douglas F. Easton, Ali Amin Al Olama, Zsofia  Kote-Jarai, Sara Benlloch (PRACTICAL), Georgia Chenevix-Trench, Antonis Antoniou, Lesley McGuffog, Fergus Couch and Ken Offit (CIMBA), Joe Dennis, Alison M. Dunning, Andrew Lee, and Ed Dicks, Craig Luccarini and the staff of the Centre for Genetic Epidemiology Laboratory, Javier Benitez, Anna Gonzalez-Neira and the staff of the CNIO genotyping unit, Jacques Simard and Daniel C. Tessier, Francois Bacot, Daniel Vincent, Sylvie LaBoissière and Frederic Robidoux and the staff of the McGill University and Génome Québec Innovation Centre, Stig E. Bojesen, Sune F. Nielsen, Borge G. Nordestgaard, and the staff of the Copenhagen DNA laboratory, and Julie M. Cunningham, Sharon A. Windebank, Christopher A. Hilker, Jeffrey Meyer and the staff of Mayo Clinic Genotyping Core Facility

Funding for the iCOGS infrastructure came from: the European Community's Seventh Framework Programme under grant agreement n° 223175 (HEALTH-F2-2009-223175) (COGS), Cancer Research UK (C1287/A10118, C1287/A 10710, C12292/A11174, C1281/A12014, C5047/A8384, C5047/A15007, C5047/A10692), the National Institutes of Health (CA128978) and Post-Cancer GWAS initiative (1U19 CA148537, 1U19 CA148065 and 1U19 CA148112 - the GAME-ON initiative), the Department of Defence (W81XWH-10-1-0341), the Canadian Institutes of Health Research (CIHR) for the CIHR Team in Familial Risks of Breast Cancer, Komen Foundation for the Cure, the Breast Cancer Research Foundation, and the Ovarian Cancer Research Fund.

**KORA:** The KORA research platform (KORA, Cooperative Health Research in the Region of Augsburg) was initiated and financed by the Helmholtz Zentrum München - German Research Center for Environmental Health which is funded by the German Federal Ministry of Education and Research and by the State of Bavaria. Furthermore, KORA research was supported within the Munich Center of Health Sciences (MC Health), Ludwig-Maximilians-Universität, as part of LMUinnovativ.

**Lothian Birth Cohort 1936:** We thank the cohort participants and team members who contributed to this study. Phenotype collection was supported by Research Into Ageing (continues as part of Age UK The Disconnected Mind project). Genotyping was funded by the UK Biotechnology and Biological Sciences Research Council (BBSRC). The work was undertaken by The University of Edinburgh Centre for Cognitive Ageing and Cognitive Epidemiology, part of the cross council Lifelong Health and Wellbeing Initiative (G0700704/84698). Funding from the BBSRC, Engineering and Physical Sciences Research Council (EPSRC), Economic and Social Research Council (ESRC), and MRC is gratefully acknowledged.

**ORCADES:** ORCADES was supported by the Chief Scientist Office of the Scottish Government, the Royal Society and the European Union framework program 6 EUROSPAN project (contract no. LSHG-CT-2006-018947). DNA extractions were performed at the Wellcome Trust Clinical Research Facility in Edinburgh. We would like to acknowledge the invaluable contributions of Lorraine Anderson and the research nurses in Orkney, the administrative team in Edinburgh and the people of Orkney.

Raine: The authors are grateful to the Raine Foundation, to the Raine Study participants and their families, and to the Raine Study research staff for cohort coordination and data collection. The authors gratefully acknowledge the NH&MRC for their long term contribution to funding the study over the last 20 years and also the following Institutions for providing funding for Core Management of the Raine Study:

- The University of Western Australia (UWA)
- Raine Medical Research Foundation
- UWA Faculty of Medicine, Dentistry and Health Sciences
- The Telethon Institute for Child Health Research
- Women and Infants Research Foundation

The authors gratefully acknowledge the assistance of the Western Australian Genetic Epidemiology Resource and the Western Australian DNA Bank (both National Health and Medical Research Council of Australia National Enabling Facilities). The authors also acknowledge the support of the National Health and Medical Research Council of Australia (Grant ID 572613 and ID 003209) and the Canadian Institutes of Health Research (Grant ID 166067). We gratefully acknowledge the assistance of the Wind Over Water Foundation, the Telethon Institute for Child Health Research, and the Raine Medical Research Foundation of the University of Western Australia.

**SASBAC:** The SASBAC study was supported by funding from the Agency for Science, Technology and Research of Singapore (A*STAR), the United States National Institute of Health (NIH) and the Susan G. Komen Breast Cancer Foundation. E.I., S.H. and K.K. were supported by grants from the Swedish Research Council, the Swedish Heart-Lung Foundation, and the Swedish Foundation for Strategic Research while working with this article.

**STG:** The Ministry for Higher Education; The Swedish Research Council (M-2005-1112); GenomEUtwin (EU/QLRT-2001-01254; QLG2-CT-2002-01254); NIH DK U01-066134; The Swedish Foundation for Strategic Research (SSF); Heart and Lung foundation no. 20070481

# Replication cohort descriptions

**ALSPAC:** The Avon Longitudinal Study of Parents and Children (ALSPAC) is a prospective population-based study that recruited a cohort of 14,541 pregnancies resident in the South West of England with expected dates of delivery 1^st^ April 1991 to 31^st^ December 1992. A total 13,678 singleton live born infants resulted from these pregnancies. The cohort have been followed-up since birth with questionnaires and regular direct clinical assessment. Full details of the cohort have been previously published (REFS 1 & 2 below) and are also available on the study website (<http://www.alspac.bris.ac.uk>.). Ethical approval for this study was obtained from the ALSPAC Law and Ethics Committee and the Local National Health Service Research Ethics Committee, and all participants provided written informed consent.

Age at menarche on these participants has been assessed prospectively via questionnaires completed annually since age 8 but the main care-giver and/or the child/young woman, which included questions about whether menstruation had started and if so at what age.

A total of 9,912 ALSPAC children were genotyped using the Illumina HumanHap550 quad genome-wide SNP genotyping platform by 23andMe subcontracting the Wellcome Trust Sanger Institute, Cambridge, UK and the Laboratory Corporation of America, Burlington, NC, USA. Individuals were excluded from further analysis on the basis of having incorrect sex assignments; minimal or excessive heterozygosity (<0.320 and >0.345 for the Sanger data and <0.310 and >0.330 for the LabCorp data); disproportionate levels of individual missingness (>3%); evidence of cryptic relatedness (>10% IBD) and being of non-European ancestry (as detected by a multidimensional scaling analysis seeded with HapMap 2 individuals, EIGENSTRAT analysis revealed no additional obvious population stratification and genome-wide analyses with other phenotypes indicate a low lambda). SNPs with a minor allele frequency of <1% and call rate of <95% were removed. Furthermore, only SNPs which passed an exact test of Hardy–Weinberg equilibrium (P >5 × 10^-7^) were considered for analysis. The resulting data set consisted of 8,365 individuals and 500527 SNPs. Of these 8365 individuals XXX had data on age at menarche and were included in the analyses here.

References:

1. Fraser A, Macdonald-Wallis C, Tilling K, Boyd A, Golding J, Davey Smith G, Henderson J, Macleod J, Molloy L, Ness A, Ring S, Nelson SM, Lawlor DA. Cohort Profile: The Avon Longitudinal Study of Parents and Children: ALSPAC mothers cohort. *International Journal of Epidemiology* 2012; doi:10.1093/ije/dys066.
2. Boyd A, Golding J, Macleod J, Lawlor DA, Fraser A, Henderson J, Molloy L, Ness A, Ring S, Davey Smith G. Cohort Profile: The ‘Children of the 90s’; the index offspring of The Avon Longitudinal Study of Parents and Children (ALSPAC). *International Journal of Epidemiology* 2012; doi:10.1093/ije/dys064. (Commissioned and reviewed cohort study description)

**BOGALUSA HEART STUDY (BHS):** Between 1973 and 2010, 9 cross-sectional surveys of children aged 4-17 years and 10 cross-sectional surveys of adults aged 18-48 years, who had been previously examined as children, were conducted for CVD risk factor examinations in Bogalusa, Louisiana. Collection of age at menarche information in the BHS has been previously described [[1](file:///C:\Documents%20and%20Settings\am264\Local%20Settings\Temporary%20Internet%20Files\Content.Outlook\ZIKU428O\Cohort%20Descriptions%203_18_2013_BHS.docx#_ENREF_1)]. Briefly, girls in the 3^rd^ grade and up were interviewed individually about menstrual history by a registered nurse during the collection of anthropometric measures, health habits, and cardiovascular risk factors. In the ongoing Longitudinal Aging Study funded by NIH and NIA since 2000, there are 1,202 subjects who have been examined 4-14 times from childhood to adulthood and have DNA available for GWA genotyping. Participants were genotyped as previously described [[2](file:///C:\Documents%20and%20Settings\am264\Local%20Settings\Temporary%20Internet%20Files\Content.Outlook\ZIKU428O\Cohort%20Descriptions%203_18_2013_BHS.docx#_ENREF_2)] using the Illumina Human610 Genotyping BeadChip. Based on the analysis of identity-by-state (IBS) sharing from whole genome genotyping data, we focus on a subset of 343 genotyped women who are of European ancestry, unrelated, and have information about the onset of menarche.

1. Wattigney WA, Srinivasan SR, Chen W, Greenlund KJ, Berenson GS (1999) Secular trend of earlier onset of menarche with increasing obesity in black and white girls: the Bogalusa Heart Study. Ethnicity & disease 9: 181-189.

2. Smith EN, Chen W, Kahonen M, Kettunen J, Lehtimaki T, et al. (2010) Longitudinal genome-wide association of cardiovascular disease risk factors in the Bogalusa heart study. PLoS genetics 6.

**CoLaus**: A population-based study to investigate the epidemiology and genetic determinants of cardiovascular risk factors and metabolic syndrome. The cohort is a random sample of the Lausanne population aged between 35 and 75. Age at menopause was self-reported some time between 2003 and 2006. Available sample size is 1013 with mean sample age 61 and mean age at menopause 50 years.

[BMC Cardiovasc Disord.](http://www.ncbi.nlm.nih.gov/pubmed/18366642) 2008 Mar 17;8:6. doi: 10.1186/1471-2261-8-6.

**CROATIA VIS, KORCULA and SPLIT**: The VIS study, Croatia, is a family-based, cross-sectional study in the isolated island of Vis that included 1,056 examinees aged 18-93. The KORCULA study, Croatia, is a family-based, crosssectional study in the isolated island of Korcula that included about 965 examinees aged 18-95. The SPLIT study, Croatia, is an ongoing population-based, cross-sectional study in the Dalmatian City of Split that included about 535 examinees aged 18-95. Studies were genotyped using Illumina HAP300v1 (VIS) or Ilumina HAP370CNV (Korcula and SPLIT) and imputation for all studies was performed using MACHv1.16. Analyses were performed using R, GenABEL and ProbABEL.

**EGCUT:** The EGCUT cohort is from the population-based biobank of the Estonian Genome Project of University of Tartu. The whole project is conducted according to Estonian Gene Research Act and all participants have signed the broad informed consent (www.geenivaramu.ee and Metspalu, 2004^1^). The cohort size is 51,515 from 18 years of age and up which reflects closely the age distribution in the Estonian population, 33% male, 67% female, 83% Estonians, 14% Russians, 3% other. Subjects are recruited by the general practitioners (GP) and physicians in the hospitals were randomly selected from individuals visiting GP offices or hospitals. Computer Assisted Personal interview (CAPI) is filled during 1-2 hours at doctors office including personal data (place of birth, place(s) of living, nationality etc.), genealogical data (family history, four generations), educational and occupational history, lifestyle data (physical activity, dietary habits, smoking, alcohol consumption, women´s health, quality of life), also anthropometric and physiological measurents are taken. Subjects for the GWAS were selected randomly all over the country^2^.

1. Metspalu, A. The Estonian Genome Project. *Drug Development Research* 62, 97-101 (2004).

2. Nelis, M. et al. Genetic structure of Europeans: a view from the North-East. *PLoS One* 4, e5472 (2009).

**Genetic Epidemiology Network of Arteriopathy (GENOA):** The Genetic Epidemiology Network of Arteriopathy (GENOA) study is a community-based study of hypertensive sibships designed to investigate the genetic underpinnings of hypertension and target organ damage [1, 2]. In the initial phase of the GENOA study (Phase I: 1996-2001), all members of sibships containing ≥ 2 individuals with essential hypertension clinically diagnosed before age 60 were invited to participate, including both hypertensive and normotensive siblings (1,583 non-Hispanic whites from Rochester, MN). The diagnosis of essential hypertension was established based on blood pressure levels measured at the study visit (>140 mmHg average systolic BP or >90 mmHg average diastolic BP) or a prior diagnosis of hypertension and current treatment with antihypertensive medications. Exclusion criteria were secondary hypertension, alcoholism or drug abuse, pregnancy, insulin-dependent diabetes mellitus, or active malignancy. In the second phase of the GENOA study (Phase II: 2000-2004), 1,241 non-Hispanic white participants were successfully re-recruited to measure potential target organ damage due to hypertension. Questionnaires were used to assess age of menopause, defined as the age of last menstruation after 12 consecutive months without menstruation. Natural menopause was defined as menopause not induced by surgery, radiation, or chemotherapy.

(1) FBPP Investigators. Multi-center genetic study of hypertension: The family blood pressure program (FBPP). Hypertension 2002, 39:3-9.

(2) Daniels PR, Kardia SL, Hanis CL, Brown CA, Hutchinson R, Boerwinkle E, Turner ST, Genetic Epidemiology Network of Arteriopathy Study. Familial aggregation of hypertension treatment and control in the Genetic Epidemiology Network of Arteriopathy (GENOA) study. Am J Med 2004, 116:676-681.

**Helsinki Birth Cohort Study** (**HBCS**): Composed of 8 760 individuals born between the years 1934-44 in one of the two main maternity hospitals in Helsinki, Finland. Between 2001 and 2003, a randomly selected sample of 928 males and 1075 females participated in a clinical follow-up study with a focus on cardiovascular, metabolic and reproductive health, cognitive function and depressive symptoms. There were 908 women with valid genotype data and data on self-reported last menstrual period. All women with menopause before age 40 or after age 60 (n=5), surgical menopause before last menstrual period (n=217), hormone replacement therapy before last menstrual period (n=91), cancer before last menstrual period (n=26), and incomplete data (n=13) were removed from the analyses. After these exclusions 556 women were included in the analyses. The mean age of the participants was 61.5 years (SD=3.1) and mean age of menopause was 50.7 years (SD=3.9). Detailed information on the selection of the HBCS participants and on the study design can be found elsewhere^42-44^. Research plan of of the HBCS was approved by the Institutional Review Board of the National Public Health Insitute and all participants have signed an informed consent.

**iCOGS**: Descriptions of the individual studies and methods can be found in: Michailidou, K., P. Hall, et al. (2013). "Large-scale genotyping identifies 41 new loci associated with breast cancer risk." Nat Genet 45(4): 353-361, 361e351-352 ([http://www.ncbi.nlm.nih.gov/pubmed/23535729](https://owa.exeter.ac.uk/owa/redir.aspx?C=thL4bovgIUWT4K9O9WtrWTrPvCYKW9AIsHWgHaDkOd-fu5hpUTAE3BL5HRH6eOfxJZxadtXhT1w.&URL=http%3a%2f%2fwww.ncbi.nlm.nih.gov%2fpubmed%2f23535729)) .

**INGI-CARLANTINO:** This cohort consisted of 1417 subjects who were drawn from Carlantino, an isolated village of southern Italy. Ethics approval was obtained from the Ethics Committee of the “IRCCS-Burlo Garofolo”, the children hospital in Trieste. Written informed consent was obtained from every participant of the study. The study population had undergone clinical and instrumental evaluations between 1998 and 2005. All samples were typed with illumina 370k CNV chip (Illumina, San Diego, USA). Imputation of genotypes was carried out using the software MACH.

**INGI-FVG:** This cohort consisted of 1700 subjects drawn from the project “Genetic Park of Friuli Venezia Giulia”. This study examined 6 isolated villages in the North-east of Italy between 2008 and 2010. Ethics approval was obtained from the Ethics Committee of the “IRCCS-Burlo Garofolo”, the children hospital in Trieste. Written informed consent was obtained from every participant of the study. The study population had undergone clinical and instrumental evaluations. All samples were typed with illumina 370k CNV chip (Illumina, San Diego, USA). Imputation of genotypes was carried out using the software MACH.

**INGI – Val Borbera:** The INGI-Val Borbera population is a collection of 1664 genotyped samples collected in the Val Borbera Valley, a geographically isolated valley located within the Appennine Mountains in NorthWest Italy. The valley is inhabited by about 3000 descendants from the original inhabitants, living in 7 villages along the valley and in the mountains. The valley was inhabited by about 10,000 people in the 19th century when endogamy was >80% . Around 1930, the population started to decrease due to emigration to South America. Participants were healthy people between 18 and 102 years of age that had at least one grandfather living in the valley. Information on participants was collected during an interview using a standardized medical questionnaire. Genotyping was performed on an Illumina array 370k Quad v3 and missing data was imputed using MACH. Association testing was conducted using ProbABEL, whilst the variance explained by genetic variants was determined using the GenABEL package within R.

Traglia, M. et al. Heritability and demographic analyses in the large isolated population of Val Borbera

suggest advantages in mapping complex traits genes. PLoS One 4, e7554 (2009).

**KORA:** The KORA studies are a series of independent population-based epidemiological surveys and follow-up studies of participants living in the region of Augsburg, Southern Germany [1,2]. All participants are of German nationality identified through the registration and informed consent has been given by all participants. The studies have been approved by the local ethics committee. The present study includes data of two KORA studies: the KORA F3 study (2004/05) which is a follow-up study of KORA S3 (1994/95), as well as the KORA S4 study (1999/2001). Reproductive parameters were obtained by a personal interview by trained medical staff. For genotyping, we included 1,644 randomly selected participants of KORA F3 and 1,814 randomly selected participants of KORA S4.

Genotyping for KORA F3 was performed using the Affymetrix 500K Array Set consisting of two chips (Sty I and Nsp I). The KORA S4 samples were genotyped with the Affymetrix Human SNP Array 6.0. Hybridisation of genomic DNA was done in accordance with the manufacturer’s standard recommendations. Genotypes were determined using the BRLMM clustering algorithm (Affymetrix 500K) and the Birdseed2 clustering algorithm (Affymetrix 6.0). For quality control purposes we applied a positive control and a negative control DNA every 48 samples (KORA F3) or every 96 samples (KORA S4). On a chip level only subjects with overall genotyping efficiencies of at least 93% were included. In addition the called gender had to agree with the gender in the KORA study database. Imputation of genotypes was performed with MACH v1.0.9 (KORA F3) and MACH v1.0.15 (KORA S4) based on HapMap II CEU reference panels. The statistical analyses were performed in R.

[1]: Holle R, Happich M, Löwel H, Wichmann HE, MONICA/KORA Study Group. KORA -- a research platform for population based health research. Gesundheitswesen 2005; 67 Suppl 1: S19-S25

[2]: Wichmann H-E, Gieger C, Illig T, MONICA/KORA Study Group. Kora-gen -- resource for population genetics, controls and a broad spectrum of disease phenotypes. Gesundheitswesen 2005; 67 Suppl 1: S26-S30

**Lothian Birth Cohort 1936 (LBC1936):** The LBC1936 consists of 1,091 relatively healthy individuals assessed on cognitive and medical traits at 70 years of age. They were born in 1936, most took part in the Scottish Mental Survey of 1947, and almost all lived independently in the Lothian region of Scotland. The sample of 548 men and 543 women had a mean age 69.6 years (SD = 0.8). A full description of participant recruitment and testing can be found elsewhere (Deary et al 2011, 2007).Genotyping was performed at the Wellcome Trust Clinical Research Facility, Edinburgh. Quality control measures were applied and 1005 participants remained.

Deary, I. J. Gow, A. J., Taylor, M. D., Corley, J., Brett, C., Wilson, V., Campbell, H., Whalley, L. J., Visscher, P.M., Porteous, D. J., & Starr, J.M. The Lothian Birth Cohort 1936: a study to examine influences on cognitive ageing from age 11 to age 70 and beyond. *BMC Geriatrics* *7*, 28 (2007).

Deary, I. J., Gow, A. J., Pattie, A., & Starr, J. M. (2011). Cohort profile: The Lothian Birth Cohorts of 1921 and 1936. International Journal of Epidemiology. doi: 10.1093/ije/dyr197.

**ORCADES:** The Orkney Complex Disease Study (ORCADES) is an ongoing family-based, cross-sectional study in the isolated Scottish archipelago of Orkney. Genetic diversity in this population is decreased compared to Mainland Scotland, consistent with the high levels of endogamy historically.

Data for participants from a subgroup of ten islands were used for this analysis. Fasting blood samples were collected and over 200 health-related phenotypes and environmental exposures were measured in each individual. All participants gave informed consent and the study was approved by Research Ethics Committees in Orkney and Aberdeen.

We genotyped 318,237 SNPs for each individual using the Illumina HumanHap300 beadchip. Alleles were called in BeadStudio using Illumina cluster files. Subjects were excluded if they fulfilled any of the following criteria: genotypic call rate <97%, mismatch between reported and genotypic sex, unexpectedly low genomic sharing with first degree relatives, excess autosomal heterozygosity, or outliers identified by IBS clustering analysis. We excluded SNPs on the basis of minor allele frequency (<0.01), HWE (P<10^-5), call rate (<97%). Pregnant women were excluded from the study. MACH v1.0.15 was used to impute over 2 million SNPs from HapMap build 36. Analyses were implemented using the GenABELand ProbABEL R libraries.

**RAINE:** Recruitment of the Western Australian Pregnancy (Raine) cohort has previously been described in detail67. In brief, between 1989 and 1991 2,900 pregnant women were recruited prior to 18-weeks gestation into a randomised controlled trial to evaluate the effects of repeated ultrasound in pregnancy. Recruitment predominantly took place at King Edward Memorial Hospital (Perth, Western Australia). Ninety percent of eligible women agreed to participate in the study. Their 2,868 babies have been followed from recruitment at the average ages of one, two, three, five, eight, ten and 14. Most of the children are of Caucasian ethnicity (82% have two Caucasian parents). Genotyping was performed using the Illumina 660w quad array and imputation was performed using MACH. Association testing was performed using R (version 2.6.2).

Newnham, J.P., Evans, S.F., Michael, C.A., Stanley, F.J. & Landau, L.I. Effects of frequent ultrasound

during pregnancy: a randomised controlled trial. Lancet 342, 887-91 (1993).

**SASBAC**: The study base included all Swedish-born women between 50 and 74 years of age who were resident in Sweden between October 1993 and March 1995. During that period, virtually all breast cancer cases in Sweden were identified, and randomly selected controls, who matched the cases in 5-year age strata, were selected from the Swedish registry of the total population. Of the eligible women, 3,345 (84%) breast cancer cases and 3,454 (82%) controls participated in this initial questionnaire-based study, providing detailed information on their use of menopausal hormone therapy, their reproductive history and other lifestyle factors. From these women, a random subsample has undergone a genome-wide association scan (803 cases, 764 controls). For the present study, only controls were considered eligible^1,2^.

1. Einarsdottir, K. et al. Comprehensive analysis of the ATM, CHEK2 and ERBB2 genes in relation to breast tumour characteristics and survival: a population-based case-control and follow-up study. *Breast Cancer Res* **8**, R67 (2006).

2. Magnusson, C. et al. Breast-cancer risk following long-term oestrogen- and oestrogen-progestin-replacement therapy. *Int J Cancer* **81**, 339-44 (1999).

**SEARCH:** The SEARCH ovarian cancer study is an ongoing, population-based ovarian cancer case–control study covering the regions served by the East Anglia and West Midlands cancer registries in the UK and has been described previously. Analyses were performed in STATA 10.0.

Song, H. et al. Common variants in mismatch repair genes and risk of invasive ovarian cancer.

Carcinogenesis 27, 2235-42 (2006).

**STG:** Between the years 2004 and 2008 population wide collection of blood on 12,647 Swedish twins born 1958 or earlier was undertaken in a project called TwinGene (REF: Magnusson PK, Almqvist C, Rahman I, Ganna A, Viktorin A, Walum H, Halldner L, Lundström S, Ullén F, Långström N, Larsson H, Nyman A, Gumpert CH, Råstam M, Anckarsäter H, Cnattingius S, Johannesson M, Ingelsson E, Klareskog L, de Faire U, Pedersen NL, Lichtenstein P. The Swedish Twin Registry: establishment of a biobank and other recent developments. Twin Res Hum Genet. 2013 Feb;16(1):317-29). The study population was recruited among twins participating in the earlier Screening Across the Lifespan Twin Study (SALT) which was a telephone interview study conducted in 1998-2002. Other inclusion criteria were that both twins in the pair had to be alive and living in Sweden. Subjects were excluded from the study if they preciously declined participation in future studies or if they had been enrolled in other STR DNA sampling projects. Menarche and menopause information was collected from the SALT interview. Women who reported to have had menopause between the ages of 41 and 59 and menarche between the ages of 9 and 17 were included in the analyses, women who had undergone hysterectomy or ovariectomy were excluded. Around 10 000 of the TwinGene study participants was genotyped using the Illumina HumanOmniExpress beadchip and imputation to HapMap2 reference panel was performed using the IMPUTE 2 software. Association analysis was performed in PLINK. Robust sandwich variance estimators were used to handle the correlated data structure.
